# Defect Engineering in Laser-Induced Graphene (LIG) Through Temperature Control: A Reactive Molecular Dynamics Study

**DOI:** 10.3390/molecules30224344

**Published:** 2025-11-10

**Authors:** Sergey V. Pavlov

**Affiliations:** Joint Institute for High Temperatures of the Russian Academy of Sciences, Moscow 125412, Russia; sergey.v.pavlov@phystech.edu

**Keywords:** ReaxFF, laser-induced graphene, molecular dynamics, defect engineering

## Abstract

Scalable and low-cost graphene synthesis remains a critical challenge for applications in energy storage, sensing, and beyond. Laser-induced graphene (LIG), produced by the rapid local carbonization of polymers like polyimide using laser irradiation, offers a promising route for the one-step, scalable fabrication of porous graphene materials. This work employs reactive molecular dynamics simulations with the ReaxFF force field to investigate the temperature dependence of polyimide carbonization into LIG. We analyze the resulting structures with a focus on the formation of functional groups. Our simulations identify an optimal carbonization temperature window near 3000 K for maximizing graphene yield. Temperatures exceeding 3500 K cause a drastic reduction in six-membered carbon rings, indicative of structural degradation. Conversely, lower temperatures (2500–2750 K) decrease graphene yield but increase the concentration of carbonyl, pyrrolic, pyridinic, and nitrile functional groups. These oxygen- and nitrogen-containing groups are potentially valuable for tailoring functionalized graphene in electrochemical and sensing applications. Furthermore, the graphitization process was found to require extended simulation times (up to ∼5 ns) to reach equilibrium, underscoring the importance of timescale in modeling such processes.

## 1. Introduction

Carbon nanomaterials, particularly graphene, are pivotal in modern materials science research due to their exceptional physicochemical properties. The two-dimensional hexagonal lattice of ${\mathrm{sp}}^{2}$-hybridized carbon exhibits remarkable characteristics, including high charge carrier mobility, exceptional mechanical and chemical stability, high thermal conductivity, and high specific surface area [1,2]. These properties enable applications in flexible electronics, energy storage, catalysis, and sensing [3,4,5]. However, the practical realization of this potential critically depends on developing reproducible synthesis methods with a controlled morphology and defect structure.

Existing graphene synthesis approaches are broadly categorized into two strategies: top-down and bottom-up [6]. Top-down methods, such as the chemical oxidation of graphite followed via a reduction (Hummers’ method [7] and its variations [8,9,10,11]), as well as liquid-phase exfoliation [12], enable large-scale production in principle but introduce structural defects and functional groups that are difficult to control, significantly altering electronic properties. Alternative bottom-up methods like chemical vapor deposition (CVD) on metal catalysts or epitaxial growth on silicon carbide yield higher crystalline quality but require complex high-temperature and vacuum conditions, alongside multistage transfer processes that limit scalability [13].

Notably, contemporary research has shifted its focus from defect minimization toward their deliberate engineering, introducing the concept of defect engineering [14,15,16]. A controlled introduction of vacancies, grain boundaries, edge sites, dislocations, heteroatoms, and functional groups can directionally modify electronic structures to create materials with tailored catalytic, sensory, or energy storage properties [14,16,17,18]. For instance, even intrinsic defects like vacancies enhance electron transfer kinetics through an increased density of states at the Fermi level [19,20].

In this context, the laser-induced graphene (LIG) method, pioneered by Tour’s group in 2014 [21], represents a breakthrough alternative. This technique relies on the direct phototransformation of polymer substrates (e.g., polyimide) into three-dimensional porous graphene under infrared laser irradiation in ambient conditions. The mechanism involves three stages: (1) photothermal heating which induces polymer carbonization, (2) degassing of volatile products which forms hierarchical porosity, and (3) the reorganization of a carbon matrix into ${\mathrm{sp}}^{2}$-domains with a tunable defect density. Key advantages of LIG include single-step mask-free synthesis, computer-controlled patterning of complex architectures, and high scalability. LIG has demonstrated success in a wide array of applications [22,23], including stretchable strain sensors [24], microsupercapacitors [25], health monitoring [26], catalysts [27], and sensing [23], establishing it as a versatile platform for the defect engineering of functional nanocarbon structures.

However, the mechanism of polyimide carbonization is not yet fully understood. Reactive force field molecular dynamics [28] (ReaxFF MD) is well suited for simulating these processes, as it combines computational efficiency with the accurate representation of chemical bond formation and breaking. This method has proven effective in modeling carbon materials across a wide temperature range [29,30,31,32].

Recent improvements in C/H/O/N reactive force fields (CHON-2019 [33] and GR-RDX-2021 [34]) have opened up the possibility of simulating LIG formation using ReaxFF [35]. Recent studies have investigated the carbonization of polyimide and other precursors of laser-induced graphene (LIG) [35,36]. In these studies, the composition of gases released during polyimide carbonization has been identified, and the processes of bond breakage within the polyimide and the subsequent formation of the ${\mathrm{sp}}^{2}$ carbon network were analyzed. Lu et al. studied the polyimide pyrolysis process in the context of polymer stability, revealing the mechanism of the initial stages of the pyrolysis process and produced gases [37]. Polyimide, as the most popular precursor for laser-induced graphene, was also studied in works [38,39], including colorless polyimide (CPI) [40]. Vashisth et al. examined graphene formation from polyimide and several other commercially available polymers via ReaxFF molecular dynamics [35]. Further, ReaxFF modeling was extended to natural polymers, such as lignin and cellulose [36] and their composites [41,42], which mimic process of producing wood-derived LIG.

Our work extends this understanding by employing longer simulation times (6 ns) to reach a thermodynamically stable state, enabling a detailed, temperature-dependent analysis of the resulting graphene-like structures, with a specific focus on the evolution and classification of functional groups and defects that are critical for defect engineering.

## 2. Computational Details

All molecular dynamics (MD) simulations were performed within the framework of the ReaxFF reactive force field using the LAMMPS software package [43] (version 29Aug2024 update2). ReaxFF employs an empirical potential based on the formalism of bond order, which allows for simulating the formation and breaking of chemical bonds [28]. The total system energy is described by the following expression:

(1) $$E_{system}=E_{bond}+E_{val}+E_{tors}+E_{under}+E_{over}+E_{lp}+E_{vdW}+E_{Coulomb}$$

where the energy terms $E_{bond}$, $E_{val}$, and $E_{tors}$ represent the bond, valence angle, and torsion angle energies, respectively; these intramolecular terms are dynamically dependent on bond orders, which are recalculated at every MD step from interatomic distances. The $E_{under}$ and $E_{over}$ terms penalize energy for atomic under- and over-coordination, ensuring correct chemical reactivity and stability of reaction intermediates and radicals. The $E_{lp}$ term accounts for the energy contribution of lone-electron pairs, which is critical for molecular geometry and hydrogen bonding. Non-bonded interactions include van der Waals $E_{vdW}$ and Coulomb $E_{Coulomb}$ energies. These are calculated for all atom pairs, independent of bonding connectivity, and are shielded at a short range. Atomic partial charges are computed using the Electronegativity Equalization Method (EEM) [44].

**Figure 1:**
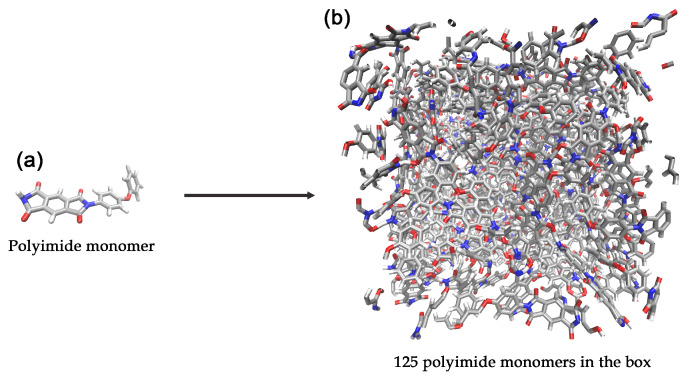
Schematic representation of (**a**) a polyimide monomer and (**b**) a simulation supercell containing 125 polyimide monomers. Color code: gray—carbon; blue—nitrogen; red—oxygen; white—hydrogen.

The initial molecular system was constructed to represent a polyimide structure. A system consisting of 125 polyimide monomers, totaling 5125 atoms, was built using the PACKMOL software package [45] (version 20.14.2). The monomers were randomly placed and oriented within a cubic simulation box with initial dimensions of 40.0 × 40.0 × 40.0 Å^3^ (see Figure 1). We used force field GR-RDX-2021, which showed high accuracy in predicting the structural and mechanical properties of ${\mathrm{sp}}^{2}$ carbon systems [34]. It was parameterized for C, H, N, and O elements, which is critical for correctly modeling the carbonization chemistry of a polyimide system.

The system was first equilibrated in the NPT ensemble and then the NVT ensemble at 300 K. The optimized box size has dimensions of approximately 39.8 × 39.8 × 39.8 Å^3^, corresponding to a mass density of 1.26 g/cm^3^. The equilibrated system was then heated from 300 K to the target pyrolysis temperatures (2500, 2750, 3000, 3250, and 3500 K) over a period of 100 ps. Then, isothermal modeling was carried out for 6 ns at each target temperature in the NVT ensemble. The duration of the simulations was chosen to be sufficient for the pyrolysis process to approach completion, within the limits of our computational resources. The temperature was controlled using a Nose–Hoover thermostat. A timestep of 0.2 fs was used to ensure numerical stability for the fast bond-breaking events occurring at high temperatures. Three-dimensional periodic boundary conditions were applied.

## 3. Results and Discussion

### 3.1. Radial Distribution Functions

First, we calculated radial distribution functions (RDFs) between all pairs of atoms present in the system to track the time evolution of RDFs, as follows: (1) relaxed polyimide at 300 K; (2) averaged over 1 ns immediately after the 100 ps heating process; and (3) for each 1 ns interval averaged during the carbonization process at different temperatures.

**Figure 2:**
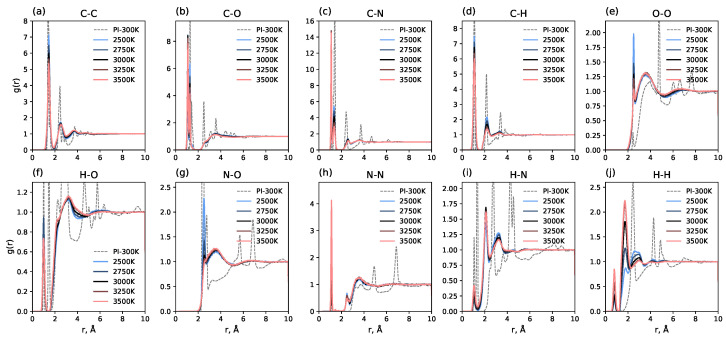
Radial distribution functions (RDFs) for different atom pairs in the system. Dashed lines show RDFs for polyimide at 300 K; solid lines show RDFs averaged over 1 ns after the 100 ps heating process to the specified carbonization temperature.

Figure 2 shows RDFs for each pair of atoms after the heating process at different carbonization temperatures in comparison with radial distribution functions for polyimide at 300 K and atmospheric pressure (indicated by dashed lines). It can be seen that the 100 ps heating process to all investigated temperatures (from 2500 K to 3500 K) greatly changes the radial distribution functions, indicating the destruction of the initial polyimide structure at these temperatures. The long-range order observed in polyimide vanishes entirely. New short-range order peaks emerge, indicating the formation of a new, more amorphous structure. The positions of these peaks are temperature-independent, but their amplitudes are sensitive to the pyrolysis temperature. Notably, the RDF for the N-N pair (Figure 2) shows a distinct peak at approximately 1.2 Å, corresponding to the N-N chemical bond; the magnitude of this peak is highly dependent on temperature. This is because molecular nitrogen (N_2_) formation is promoted at higher temperatures; at lower temperatures, nitrogen tends to remain in non-molecular forms. A similar effect is observed for the H-H pair: higher temperatures facilitate the formation of molecular hydrogen. Conversely, for N-O and O-O pairs, lower temperatures lead to an increase in the amplitude of the first peaks. Another interesting feature is the presence of double peaks for C-O and C-N pairs. This indicates the presence of different bond orders in the system, which have different equilibrium bond lengths.

**Figure 3:**
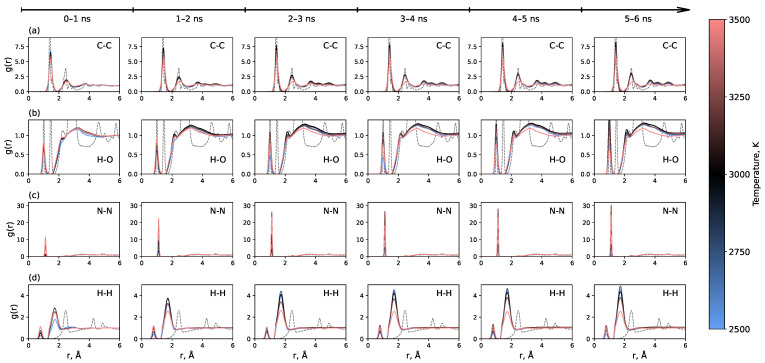
Time evolution of radial distribution functions for (**a**) C-C, (**b**) H-O, (**c**) N-N, and (**d**) H-H pairs throughout the entire 6 ns simulation trajectory after heating. Data are averaged over consecutive 1 ns intervals.

Figure 3 shows the time evolution of key RDFs throughout the simulation. The most pronounced effect is observed for the C-C RDF, which demonstrates the gradual formation of a long-range order, signaling the development of an sp^2^ carbon network. The temperature of 3000 K yields the most pronounced long-range order, while the system at 3500 K behaves distinctly differently, failing to develop a long-range order. The RDFs for H-H, N-N, and H-O also evolve over time, with a noticeable increase in the N-N bond peak. The fact that the RDFs continue to change for up to 5–6 ns indicates that the graphene formation process is relatively slow on the typical timescale of molecular dynamics simulations. This must be considered when modeling polymer carbonization, as many previous studies used simulation times shorter than 2 ns [35,36,38,40,41,42]. RDFs for all other pairs of atoms are presented in Figure S1. They show a less pronounced dependence on time.

**Figure 4:**
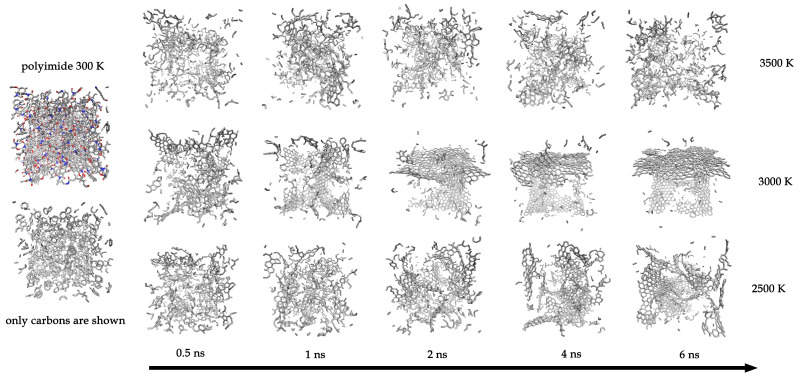
**Left panel**: Visualization of the initial polyimide system at 300 K showing all atoms and bonds (**top**) and only C-C bonds (**bottom**). Horizontal panels: Snapshots of the systems after 0.5, 1, 2, 4, and 6 ns of carbonization at three representative temperatures: 2500 K, 3000 K, and 3500 K.

Figure 4 provides a visual representation of the simulation cell. It shows the polyimide at 300 K (all atom types and C-C bonds) and subsequent snapshots at different times for three representative temperatures: 2500 K, 3000 K, and 3500 K. Visually, the system at 3000 K forms the most continuous and least defective graphene-like sheet. At 2500 K, a more fragmented structure is observed; at 3500 K, no continuous graphene layer forms, and only small, defective fragments are present. Corresponding visualizations for all temperatures are provided in the Supporting Information (see Figure S2).

### 3.2. Cluster Analysis

Using defined thresholds of bond lengths for each of pair from RDFs, we employed cluster analysis based on graphs. For each frame of the trajectory, a graph of connections between atoms is constructed based on distances and threshold values for pairs of elements. The values of thresholds were determined as the minima of the corresponding RDFs and are listed in Table 1. Connected components of the graph (clusters) are identified as separate molecular structures, which further were classified and counted.

Next, the clusters were classified, and the most frequently occurring ones were identified. Moreover, we monitored changes in their numbers. Among these, clusters were identified as molecules (CO, ${\mathrm{CO}}_{2}$, $H_{2}O$, $O_{2}$, $H_{2}$, $N_{2}$), the original polyimide monomers, and various fragments, which were divided into groups based on the number of carbon atoms in their composition ($C_{3}$–$C_{4}$, $C_{5}$–$C_{6}$, $C_{7}$–$C_{15}$, $C_{16}$–$C_{17}$, $C_{18}$–$C_{21}$).

**Figure 5:**
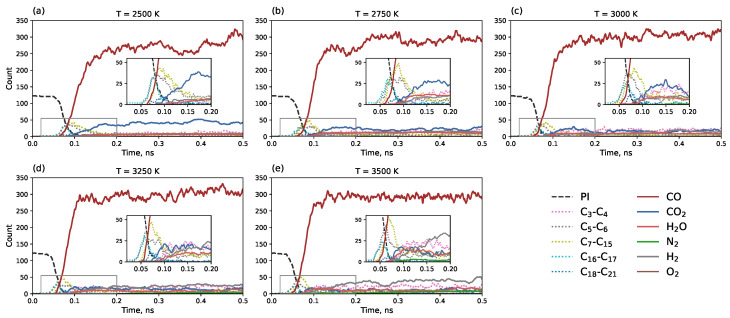
Time dependence of component counts during the first 0.5 ns of simulation (0.1 ns heating + 0.4 ns isothermal modeling). The black dashed line represents polyimide monomers. Separate counts are shown for small molecules (CO, ${\mathrm{CO}}_{2}$, $H_{2}O$, $N_{2}$, $H_{2}$, $O_{2}$) and carbon clusters grouped by size: $C_{3}$–$C_{4}$, $C_{5}$–$C_{6}$, $C_{7}$–$C_{15}$, $C_{16}$–$C_{17}$, $C_{18}$–$C_{21}$ depending on the temperature: (**a**) 2500 K, (**b**) 2750 K, (**c**) 3000 K, (**d**) 3250 K, and (**e**) 3500 K.

Figure 5 illustrates the system dynamics during the first 0.5 ns. The number of polyimide monomers (dashed black line) decreases rapidly upon heating. The first clusters to form are C_16_–C_17_ and C_5_–C_6_ fragments. This is consistent with one of the most energetically favorable polyimide pyrolysis mechanisms, where the polyimide monomer, being a C_22_ molecule, cleaves at the C-O bond, as reported previously [36]. Further, a sharp peak of C_7_–C_15_ fragments is observed, indicating further cleavage of the C-N bond, which is also an energetically favorable route [36]. Simultaneously, an intensive release of CO gas is observed. An interesting temperature-dependent feature is the higher quantity of CO_2_ molecules at lower temperatures, although their number remains significantly lower than that of CO. Carbon monoxide has been previously reported as the main gaseous product of pyrolysis for polyimide and several other polymers [35,40], as well as for lignin and cellulose [36] and lignin-rich wood [41]. At the highest temperature of 3500 K, an increased formation of H_2_ molecules is noted.

**Figure 6:**
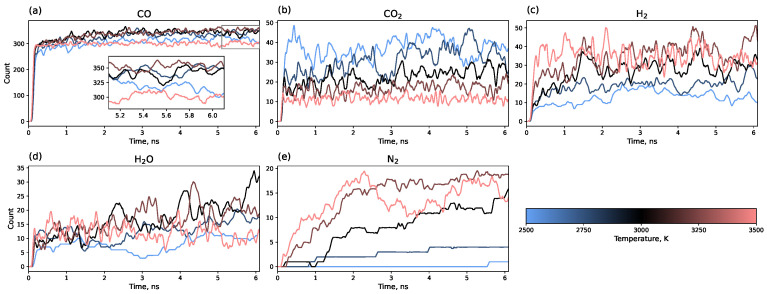
Time evolution of small molecule counts: (**a**) CO, (**b**) ${\mathrm{CO}}_{2}$, (**c**) $H_{2}$, (**d**) $H_{2}O$, (**e**) $N_{2}$ along the entire simulation trajectory (0.1 ns heating + 6 ns isothermal modeling).

Figure 6 shows the evolution of small molecule counts over the entire simulation trajectory. An increase in the number of H_2_ and N_2_ molecules with a rising temperature is evident. Although H_2_ is released almost immediately after heating and then fluctuates around an equilibrium value, the number of N_2_ molecules requires 3–5 ns to reach equilibrium. The quantities of CO_2_, CO, and H_2_O also correlate with temperature: the amount of CO_2_ decreases with increasing annealing temperature. The maximum yield of CO and H_2_O is observed at 3000 K, with lower quantities at both higher and lower temperatures.

**Figure 7:**
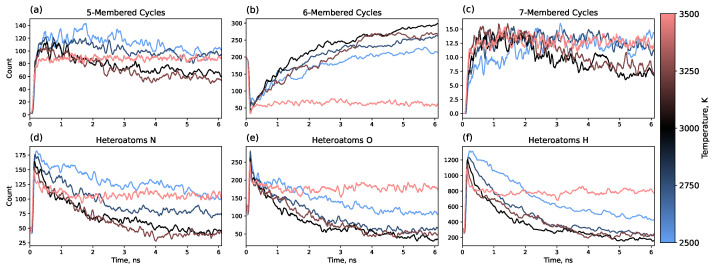
Time dependence of (**a**–**c**) carbon ring counts (5-, 6-, and 7-membered rings, respectively) in the whole system and (**d**–**f**) heteroatom content (nitrogen, oxygen, and hydrogen, respectively) within the graphene-like clusters formed during the annealing process.

In addition, 5-, 6-, and 7-membered carbon rings were counted throughout the trajectory. Rings were counted in the whole system before dividing the system into clusters for a convenient comparison with previously published data. Therefore, initially before heating, 6-member cycles are present as they are in polyimide monomers (see Figure 1 and Figure 7b). The heating process eliminates approximately three-quarters of the 6-membered cycles, which are then slowly restored to form a graphene matrix. This is in good agreement with previously published results, showing a similar reduction in the number of 6-membered cycles and recovery to approximately the initial number after 1.25 ns for polyimide polymers [35].

Simultaneously, the formation of 5- and 7-membered rings is observed, with a higher number of 5-membered rings. Our results demonstrate that, over time, a fraction of these 5- and 7-membered rings undergo a structural transition into 6-membered rings, thereby reducing the overall defect density. This structural evolution effectively represents a healing mechanism within the graphene lattice. The most effective healing occurs at 3000 K, where the numbers of 5- and 7-membered rings decrease by 40–50% from their maximum values over 6 ns.

The observed healing is consistent with experimental reports under various conditions [46,47,48] and has been reproduced in simulations [29,49]. Furthermore, these findings align with reports of self-healing behavior in graphene subjected to multiple cycles of laser irradiation [38,47].

### 3.3. Analysis of Heteroatoms in Graphene

Next, we analyzed graphene-like clusters separately. These were defined as clusters comprising at least 50 atoms, with a minimum of 50% carbon content and at least 10% of atoms having three bonded neighbors. For these clusters, we determined the number of heteroatoms and categorized their functional groups.

The criteria for graphene-like clusters were set quite leniently in order to analyze such clusters from the very beginning of the simulation. In practice, the first 0.5–1 ns yields quite defective structures; however, after 2–3 ns of modeling, there is typically just 1 or 2 such clusters in the system. To illustrate this, Figure S3 provides information about the number of graphene-like clusters along the MD trajectory and the size of the largest one. It can be seen that there is an initial surge in the number of such clusters during the heating process, but the system converges to several (1–3) large clusters quite quickly. It is noticeable that at a temperature of around 3000 K, the system reaches the point of having exactly one cluster after approximately 1 ns. It is also noteworthy that the size of the large clusters gradually decreases during annealing, which is consistent with the healing process. For a temperature of 3500 K, healing does not occur; the size of the cluster fluctuates around 3000 atoms. At 3000 K and 3250 K, the system shows a decrease in the size of the biggest cluster from more than 3000 atoms to approximately 2000 atoms.

Figure 7d–f shows the overall number of heteroatoms within graphene-like clusters during the simulation. It can be seen that the number of heteroatoms decreases during annealing, with the characteristic time of this process coinciding with the characteristic time of the formation of 6-membered rings. Furthermore, the temperature dependence also reveals a correlation between defects in terms of both internal disruptions of the sp^2^ network and the presence of heteroatoms. Overall, 3000 K shows the highest number of 6-membered rings in the system and, simultaneously, the lowest number of heteroatoms.

**Figure 8:**
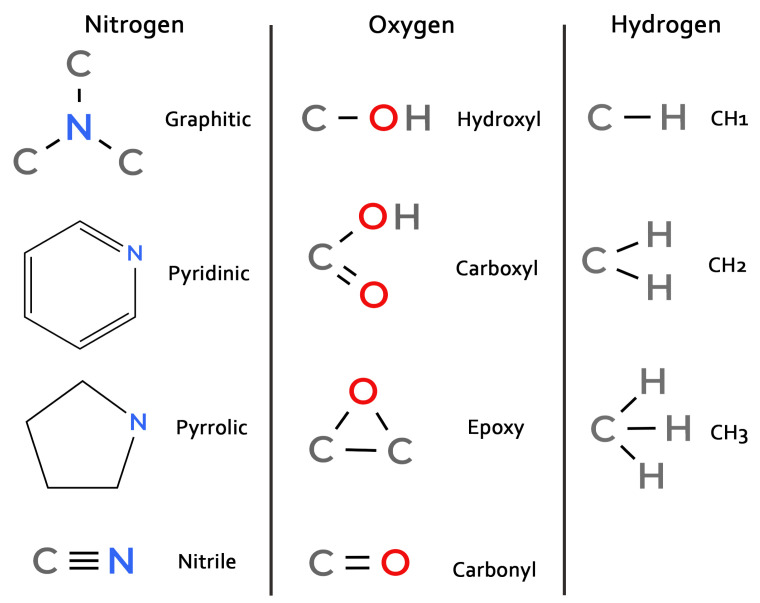
Schemes of classified functional groups.

Since defect engineering requires knowledge of not only the type of heteroatoms but also their specific functional groups, which critically influence catalytic and sensory properties [5,27], we conducted a detailed analysis of each N, O, and H heteroatom within the graphene-like clusters. Heteroatoms were classified into the functional groups depicted in Figure 8. Atoms not matching any defined group were labeled as “other”; their population was found to be negligible compared to the classified groups.

The classification of nitrogen defects deserves special attention, as pyrrolic nitrogen in the 5-membered ring is typically bonded to hydrogen. However, in high-temperature simulations, nitrogen in the 5-membered ring is often not bonded to hydrogen. Therefore, the pyrrolic and pyridinic functional groups were defined by ring size rather than by the presence of a hydrogen bond. This is reflected in the diagram in Figure 8.

**Figure 9:**
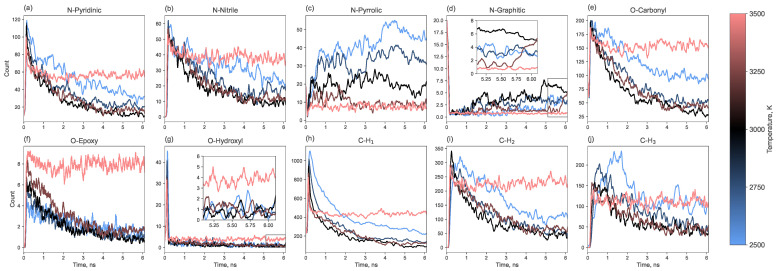
Time evolution of functional group counts (the three most abundant groups for each heteroatom type) along the entire simulation trajectory.

Figure 9 presents the dynamics of the most frequent functional groups (pyridinic, nitrile, pyrrolic, and graphitic for nitrogen; carbonyl, epoxy, and hydroxyl for oxygen; and C-H_1_, C-H_2_, and C-H_3_ for hydrogen). The most common nitrogen-containing group is pyridinic nitrogen, followed by nitrile and pyrrolic groups and then graphitic nitrogen. The populations of pyridinic and nitrile groups decrease over time. In contrast, the content of pyrrolic and graphitic nitrogen increases. The growth of graphitic nitrogen at the final stage of pyrolysis can be explained by the healing of graphene clusters: pyridinic, pyrrolic or nitrile groups located at the edges can become incorporated into the graphene matrix as the structure heals, resulting in the nitrogen atom being “captured” in a graphitic configuration. The number of pyrrolic nitrogen atoms probably grows by combining nitrogen with 5-membered carbon rings, the number of which is reduced during the annealing.

The populations of oxygen- and hydrogen-containing groups generally decrease over time. The most prevalent oxygen functional group is carbonyl, with a smaller number of epoxy groups. Hydroxyl groups appear early in the process but almost completely disappear later. Hydrogen is primarily found in C-H groups, less frequently in C-H_2_, and rarely in CH_3_ groups.

Collectively, the dynamic evolution of functional groups, particularly the conversion of pyridinic and nitrile nitrogen to pyrrolic and graphitic nitrogen over time (Figure 9), illustrates the complex interplay between defect formation and annealing during the carbonization process. This suggests that the final defect state is not only temperature-dependent but also influenced by the duration of the annealing process.

## 4. Final State Characterization

**Figure 10:**
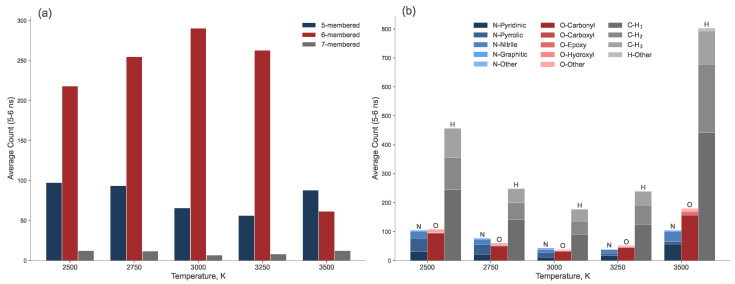
Equilibrium quantities of (**a**) carbon rings and (**b**) heteroatoms (categorized by functional group type) in the graphene clusters, averaged over the final 1 ns of the MD trajectory.

To summarize the properties of the resulting laser-induced graphene as a function of annealing temperature, we averaged the results over the 5–6 ns time range and presented them as bar charts, as shown in Figure 10. Panel (a) shows the final count of 5-, 6-, and 7-membered carbon rings, while panel (b) shows the heteroatom content, categorized by functional group type. The maximum number of 6-membered rings at 3000 K coincides with the minima of 5- and 7-membered rings, as well as the lowest concentration of all three types of heteroatoms. This clearly indicates that 3000 K is the optimal temperature for maximizing graphene yield while minimizing both intrinsic (ring defects) and extrinsic (heteroatom) defect densities.

The temperature directly dictates the balance between defect creation and healing. At lower temperatures (2500–2750 K), the kinetic energy is insufficient to fully reorganize the carbon lattice, resulting in higher concentration of intrinsic defects and heteroatoms. Specifically, we observe a higher yield of oxygen-containing carbonyl groups and nitrogen in pyridinic, pyrrolic and nitrile configurations (Figure 9 and Figure 10b). These nitrogen functional groups are known to enhance the electrochemical activity of carbon materials [50,51], suggesting that low-temperature LIG could be preferentially tailored for sensing or catalytic applications without the need for post-synthesis functionalization.

A comparison of the functional groups at 2750 K and 3250 K reveals a distinct shift in their distribution: at 2750 K, there is a higher concentration of nitrogen heteroatoms than oxygen heteroatoms, with the opposite being true at 3250 K. Furthermore, the number of pyrrolic groups decreases monotonically with increasing temperature. At 3500 K, the structure undergoes degradationn of the sp^2^ network, as indicated by the sharp decrease in 6-membered rings and the formation of pentagons and heptagons (Figure 10a).

## 5. Conclusions

This study employed ReaxFF molecular dynamics simulations to investigate the temperature-dependent mechanism of laser-induced graphene formation from polyimide. The results unequivocally demonstrate that the annealing temperature is a critical parameter for controlling the structure and composition of the resulting carbon material, enabling precise defect engineering.

A critical insight from this work is the slow kinetics of the process. The structural properties continue to evolve for up to 5–6 ns before reaching a steady state, highlighting that simulation times significantly longer than those commonly used are necessary to capture the system’s thermodynamic equilibrium.

A key finding is the identification of an optimal carbonization temperature near 3000 K for producing high-quality LIG. At this temperature, the system achieves the highest proportion of 6-membered carbon rings and the lowest concentration of heteroatoms (O, N, H), indicating the efficient formation of a continuous sp^2^-carbon network. Deviations from this optimum yield distinct outcomes: lower temperatures (2500–2750 K) result in incomplete carbonization, producing structures rich in functional groups (e.g., carbonyl, pyridinic, pyrrolic) that are suitable for applications such as electrochemistry. Conversely, temperatures above 3500 K cause severe structural degradation, characterized by a loss of a long-range order and an increase in 5- and 7-membered rings.

From a practical point of view, these results can guide the tailoring of LIG properties by controlling the laser parameters (e.g., power, scan speed, cooling rate, multiple lasing, defocusing) that directly influence the local pyrolysis temperature. Additionally, the type of laser and its mode of operation (pulsed or continuous) can also affect the temporal characteristics of the process, as different types of lasers have varying characteristic interaction times with the system across a wide range from $10^{-15}$ to 10^−1^ s [52].

Further experimental validation of functional group content via X-ray Photoelectron Spectroscopy (XPS), depending on pyrolysis conditions, is also desirable, as well as other characterization techniques, such as Transmission Electron Microscopy (TEM), Scanning Electron Microscopy (SEM), X-ray Diffraction (XRD), Raman and UV-Vis spectroscopy, and other methods.

In summary, these findings provide atomic-scale insights for optimizing experimental LIG fabrication. The quality and functional properties of LIG are shown to be tunable via a synthesis temperature, paving the way for the rational design of carbon materials for energy storage, sensing, and nanoelectronics.

## Figures and Tables

**Table 1:** Threshold distances for determining chemical bonds between pairs of elements.

Element Pair	Threshold (Å)
C-C	1.9
C-H	1.6
C-O	2.0
H-O	1.3
H-N	1.5
C-N	1.9
N-O	1.8
H-H	1.1
N-N	1.9
O-O	1.8

## Data Availability

The raw data supporting the conclusions of this article will be made available by the authors on request.

## Supplementary Materials

The following supporting information can be downloaded at: https://www.mdpi.com/article/10.3390/molecules30224344/s1. Figure S1: Time evolution of radial distribution functions for all pairs of atoms; Figure S2: Simulation snapshots for all carbonization temperatures; Figure S3: Number of graphene-like fragments in the systems and the sizes of the largest ones at different temperatures; Videos S1–S5: Visualizations of system’s time evolution at different temperatures.

## Abbreviations

The following abbreviations are used in this manuscript:

LIG	Laser-induced Graphene
RDF	Radial Distribution Function
MD	Molecular Dynamics
XPS	X-ray Photoelectron Spectroscopy
TEM	Transmission Electron Microscopy
SEM	Scanning Electron Microscopy
XRD	X-ray Diffraction
