# Supplementary material for: Defect Engineering in Laser-Induced Graphene (LIG) Through Temperature Control: A Reactive Molecular Dynamics Study
Source: Molecules. 2025 Nov 10;30(22):4344. doi: 10.3390/molecules30224344 (PMC12655355; doi:10.3390/molecules30224344)
Supplement: Supplementary file 1 [file molecules-30-04344-s001.zip › supporting.pdf]

Supporting Information on:

Defect Engineering in Laser-Induced Graphene (LIG) Through Temperature Control: A Reactive Molecular Dynamics Study

Sergey V. Pavlov*

*sergey.v.pavlov@phystech.edu

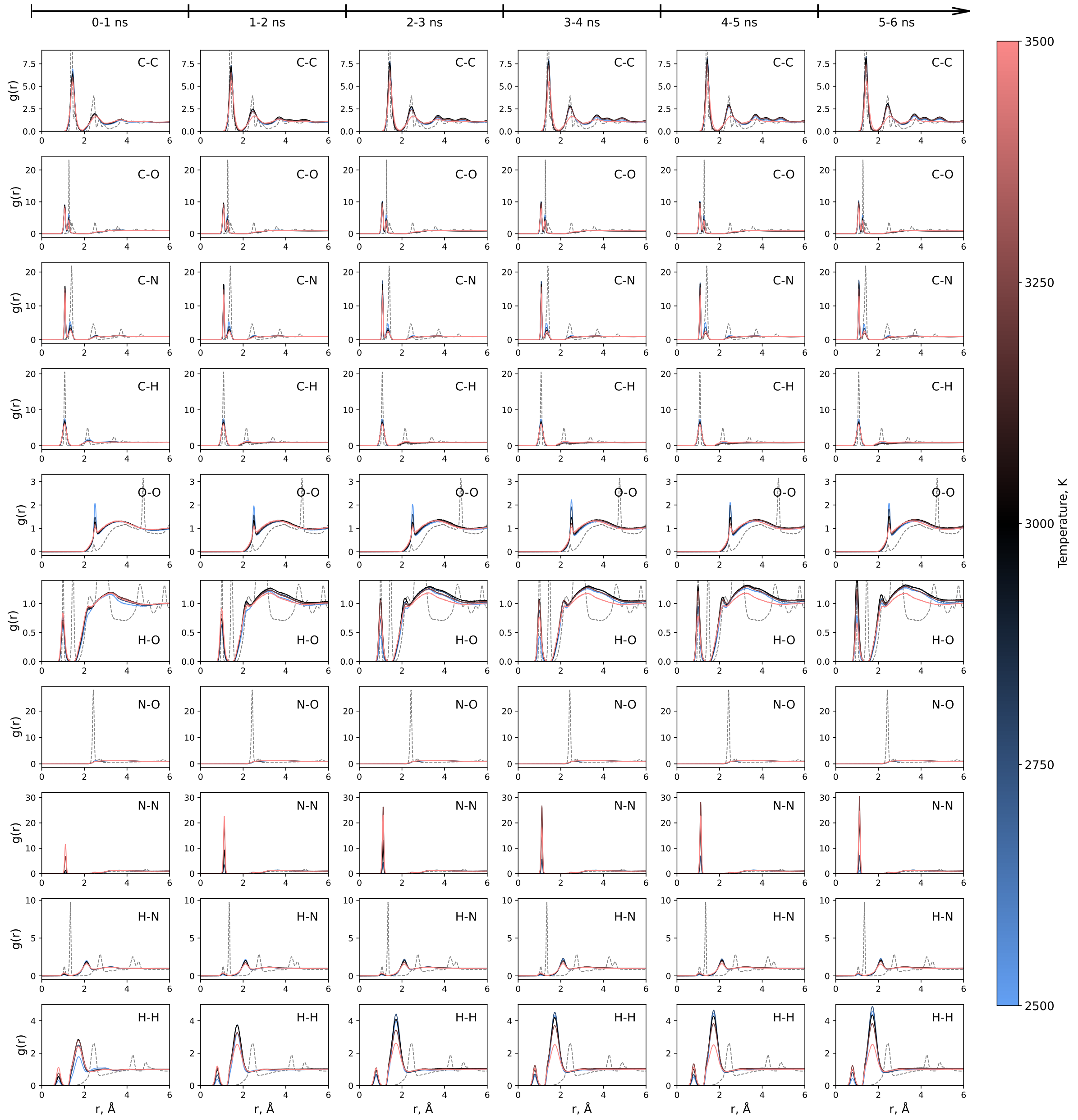

Figure S1: Time evolution of radial distribution functions (RDFs) for all pairs of atoms throughout the entire 6 ns simulation trajectory after heating. Data are averaged over consecutive 1 ns intervals. Dashed lines show RDFs for polyimide at 300 K

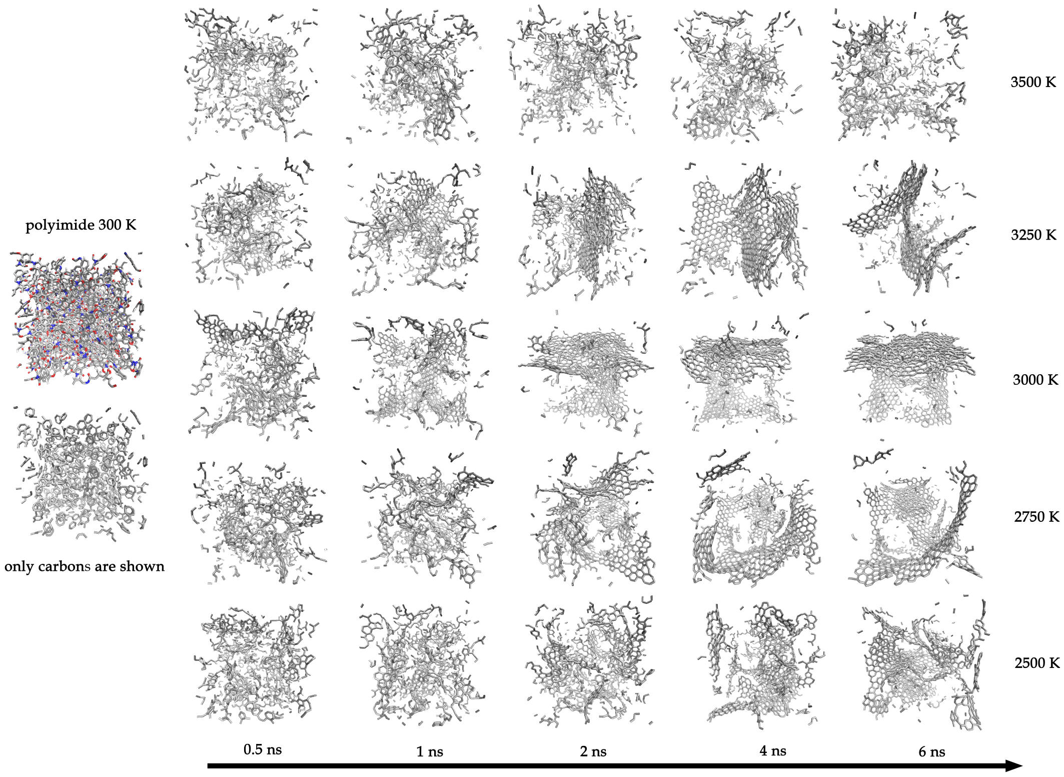

Figure S2: Simulation snapshots for all carbonization temperatures at 0.5, 1, 2, 4, and 6 ns time points.

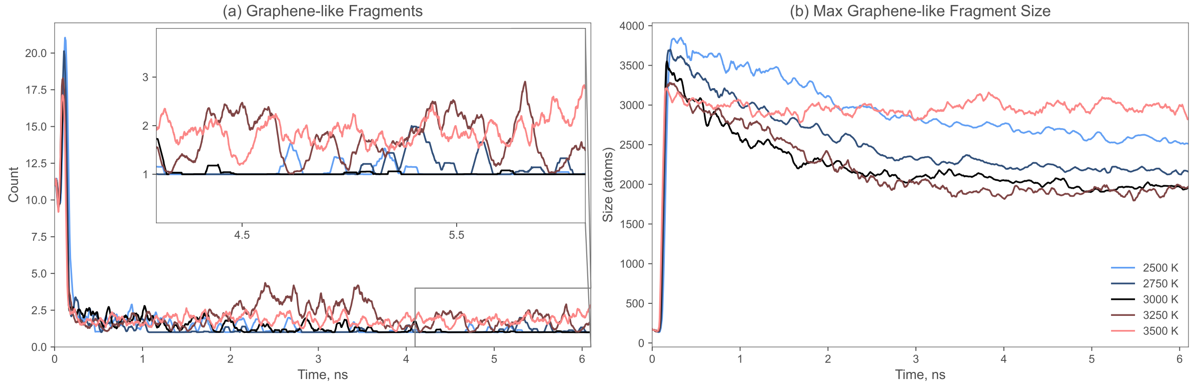

Figure S3: (a) Number of graphene-like fragments in the system at different temperatures. (b) The size of the largest graphene-like fragment.
