# Supplementary figures and images for: Defect Engineering in Laser-Induced Graphene (LIG) Through Temperature Control: A Reactive Molecular Dynamics Study
Source: Molecules. 2025 Nov 10;30(22):4344. doi: 10.3390/molecules30224344 (PMC12655355; doi:10.3390/molecules30224344)

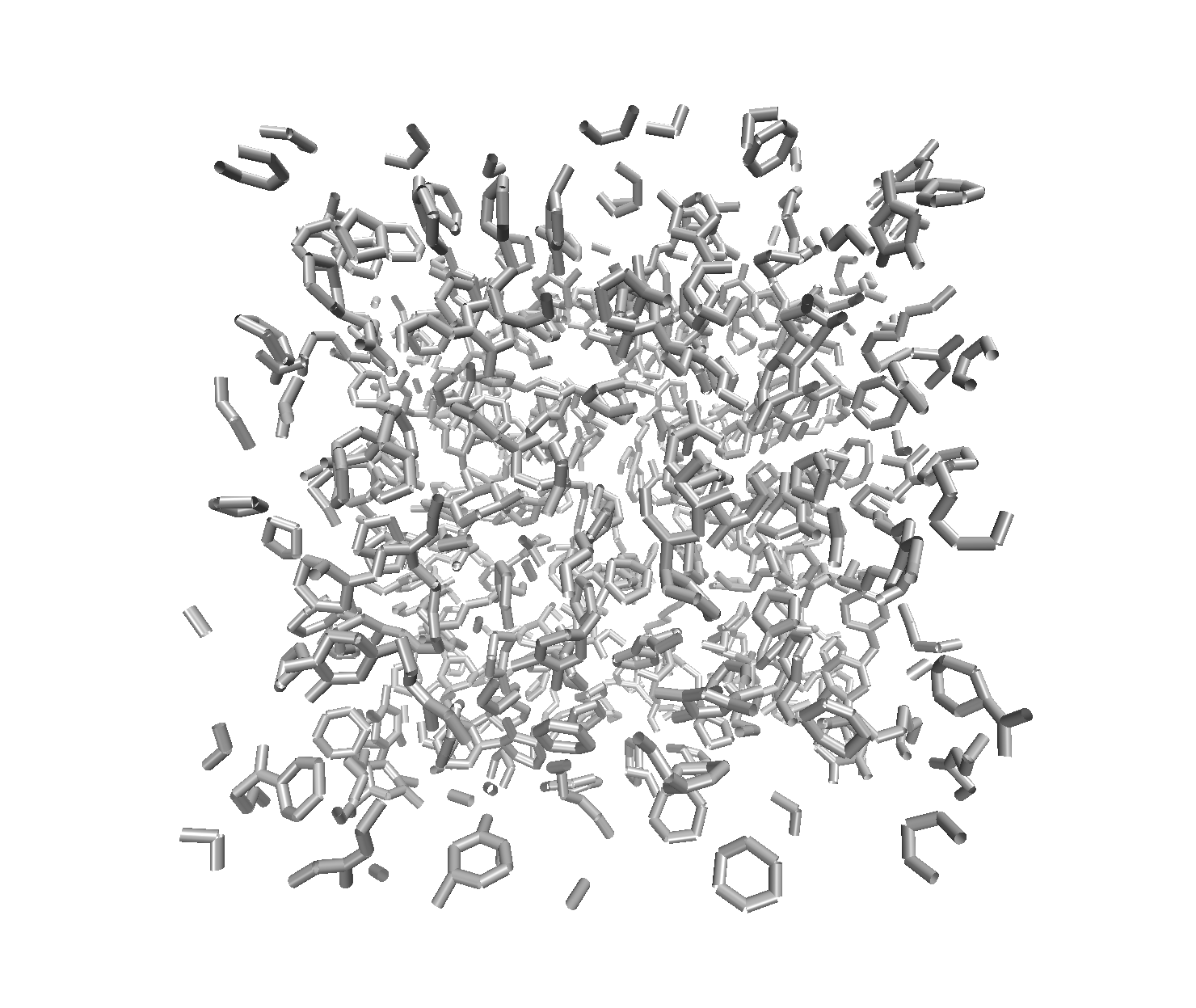

Supplement: Supplementary file 1 [file molecules-30-04344-s001.zip › Video S1. PI-2500K.gif]

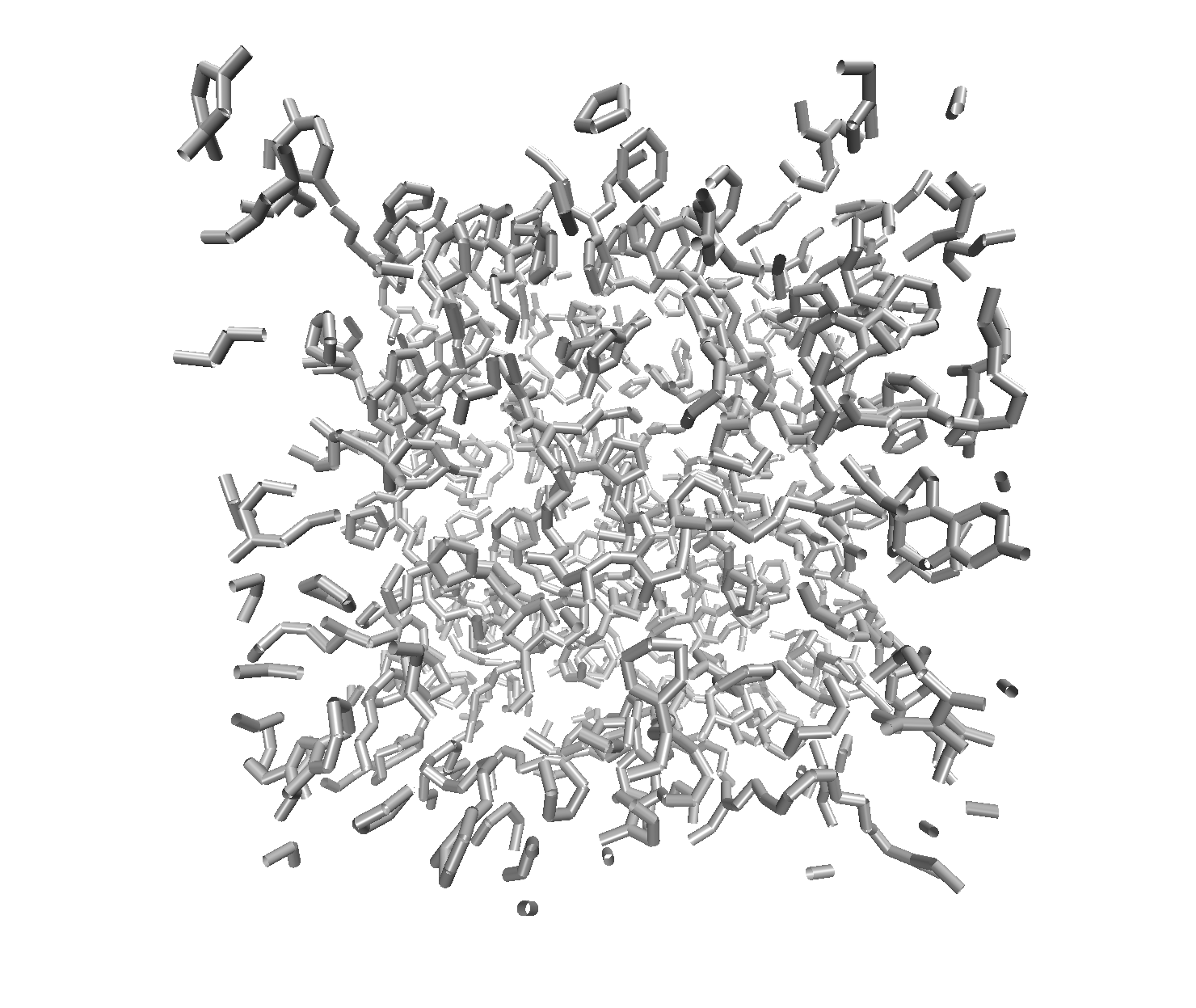

Supplement: Supplementary file 1 [file molecules-30-04344-s001.zip › Video S2. PI-2750K.gif]

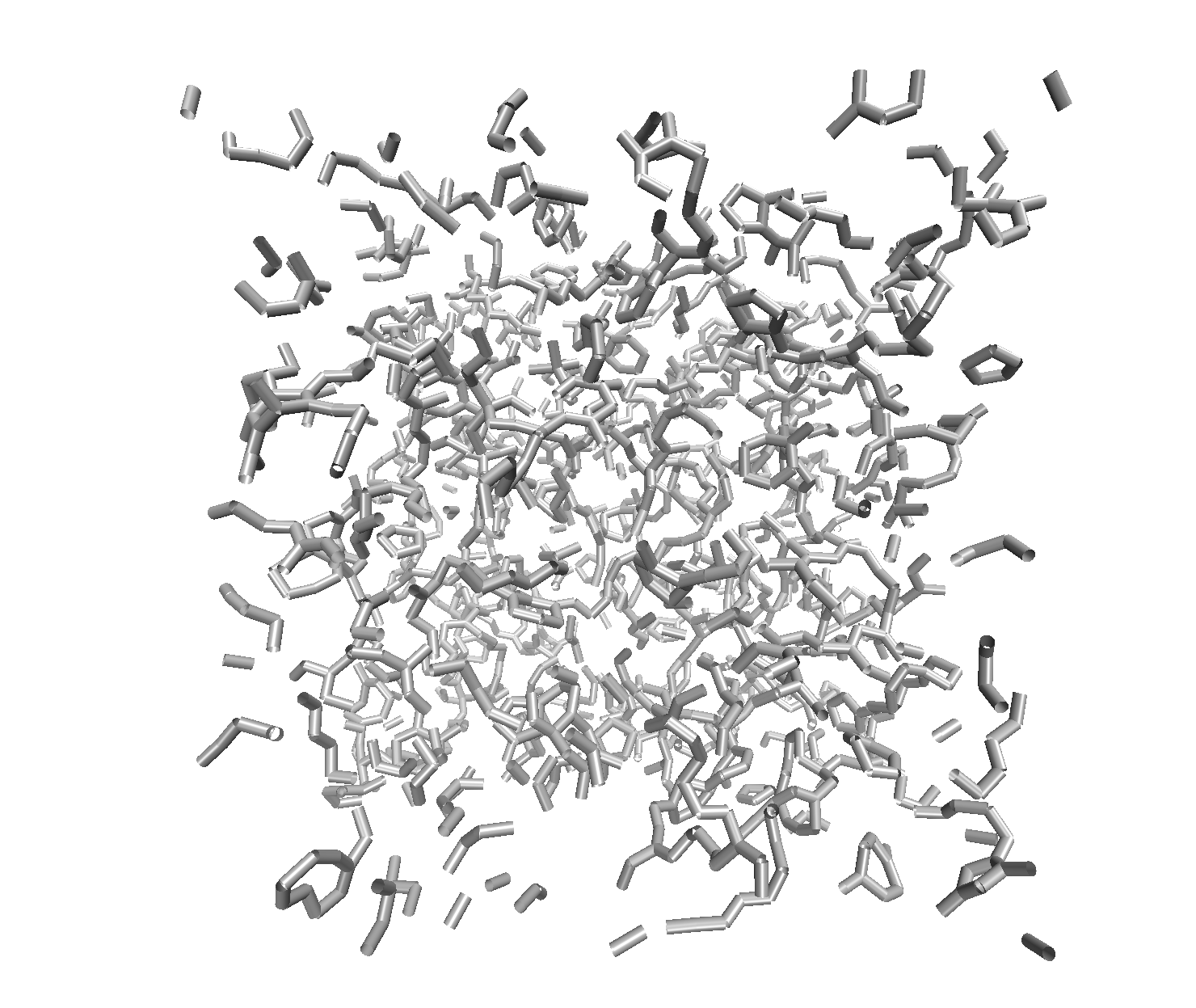

Supplement: Supplementary file 1 [file molecules-30-04344-s001.zip › Video S3. PI-3000K.gif]

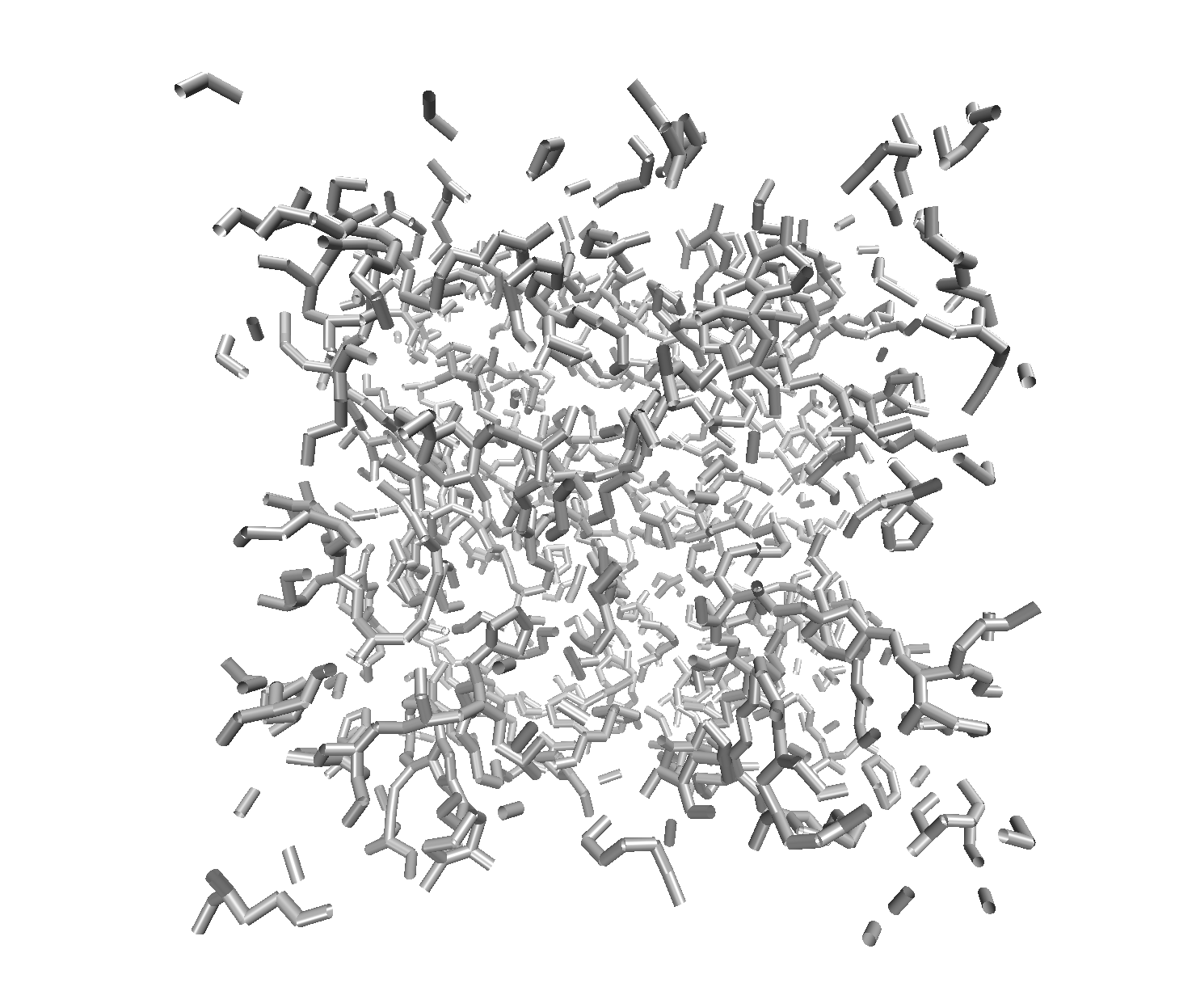

Supplement: Supplementary file 1 [file molecules-30-04344-s001.zip › Video S4. PI-3250K.gif]

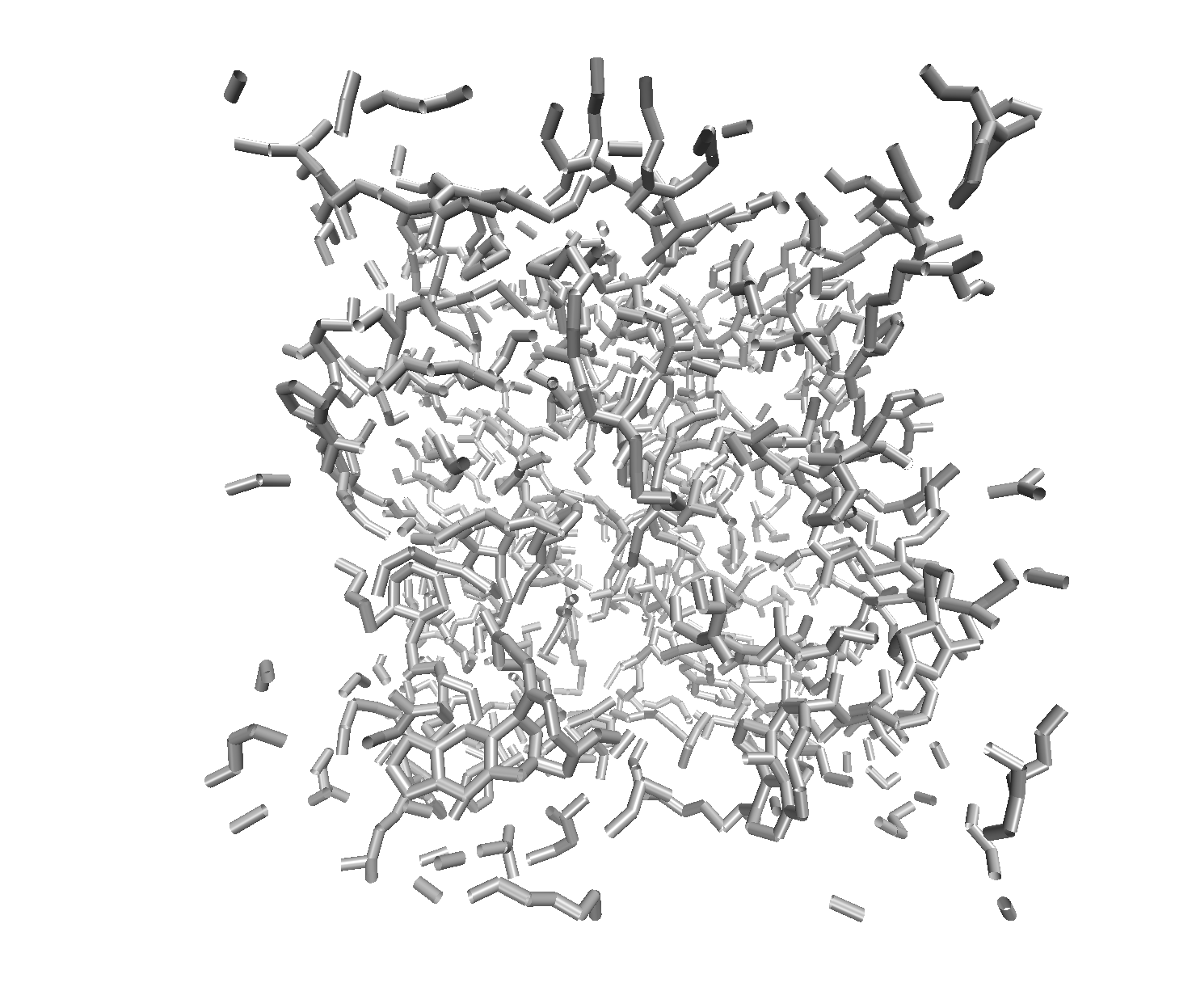

Supplement: Supplementary file 1 [file molecules-30-04344-s001.zip › Video S5. PI-3500K.gif]
